# Antinociceptive and Anti-Inflammatory Activities of* Teucrium persicum* Boiss. Extract in Mice

**DOI:** 10.1155/2015/972827

**Published:** 2015-11-16

**Authors:** Abdolhossein Miri, Javad Sharifi-Rad, Kaveh Tabrizian, Ali Akbar Nasiri

**Affiliations:** ^1^Zabol Medicinal Plants Research Center, Zabol University of Medical Sciences, Zabol, Iran; ^2^Department of Pharmacognosy, Faculty of Pharmacy, Zabol University of Medical Sciences, Zabol, Iran; ^3^Department of Pharmacology and Toxicology, Faculty of Pharmacy, Zabol University of Medical Sciences, Zabol, Iran; ^4^Department of Anesthesiology, Zabol University of Medical Sciences, Zabol, Iran

## Abstract

*Background.* Therapeutic properties of* Teucrium* species as antioxidant, antibacterial, analgesic, anticancer, diuretic, and tonic compounds have been proved earlier.* Materials and Methods*. In this study, the antinociceptive and anti-inflammatory effects of the aqueous extract of* Teucrium persicum* on chronic pain, sciatic nerve ligation as a model of neuropathic pain, and inflammatory models were investigated by formalin, hot-plate, and cotton pellet-induced granuloma models in mice, respectively.* T. persicum* aqueous extracts (100, 200, and 400 mg/kg) were orally gavaged for one week. On 8th day, the time spent and the number of lickings were recorded in formalin test. Morphine and Diclofenac were used intraperitoneally as positive controls. In sciatic nerve ligated animals, as a model of neuropathic pain, doses (100, 200, and 400 mg/kg) of* T. persicum* extract (TPE) were orally gavaged for 14 consecutive days. The analgesic effect of this extract was examined 14 days after sciatic nerve ligation using the hot-plate test. Controls received saline and Imipramine (40 mg/kg, i.p.) was used a positive control for neuropathic pain model.* Results.* In the formalin test, a week oral gavage of all TPE doses (100, 200, and 400 mg/kg) caused a significant decrease on the licking response compared to the control negative animals. In the hot-plate test, doses of 200 and 400 mg/kg showed significant analgesic effects in sciatic nerve ligated animals. Oral gavaged of TPE revealed significant analgesic effect on chronic pain in both formalin test and sciatic nerve ligated animals. The TPEs did not have any significant anti-inflammatory effects in cotton pellet-induced granuloma formation in mice.* Conclusions.* These results suggest that the aqueous extract from* T. persicum* Boiss. produced antinociceptive effects. Its exact mechanism of action still remains indistinct.

## 1. Introduction

Pain, a multidimensional sensory experience, may be associated with avoidance motor reflexes and autonomic output alterations. It could be experienced as several types such as nociceptive, inflammatory, neuropathic, and functional pain and is also generated by different neurobiological mechanisms [1, 2]. It is very important to control this alarm nociceptive pain system during clinical conditions [3]. Neuropathic pain is a consequence of somatosensory pathway legions in the peripheral or central nervous system [4]. Successful management of neuropathic pain necessitates the careful diagnosis of the cause of neuropathic pain, patient education, and the attention to coexisting nervous system disturbances [4–7]. To find a new view in management of this pain state, it is very important to understand the underlying mechanisms of neuropathic pain. Chronic nerve ligation as a model of neuropathic pain is sometimes refractory to conventional antinociceptive drugs and may lead to degeneration of A- and C-type nerve fibres [8–10]. The effectiveness of Morphine and other common used opioid analgesics has been limited by the incidence of opioid-induced adverse effects and withdrawal syndromes. Antidepressants such as amitriptyline or Imipramine have been used in last decades in management and treatment of neuropathic pain syndromes. Therefore, other therapeutic methods may play a key role in pain control strategies by targeting at the type of experienced pain.

Therapeutic properties of* Teucrium* species as antioxidant, antibacterial, analgesic, anticancer, diuretic, and tonic compounds in several kinds of disorders have been well understood [11].* Teucrium persicum* is one of the 12 species of* Teucrium* in the flora of Iran that is found in large quantities in Fars. Antinociceptive and anti-inflammatory activities of some* Teucrium* species have been well documented [12–15].

Use of herbs as a source of medicine continues to be an important component of the health care system [16–23]. The search of biologically active ingredient of plants has constantly been great interest to researcher looking for new sources of practical alternative against diseases [24–29].

Due to the reported use of other* Teucrium* species in pain and inflammatory related disorders and to open a new view in the control of these suffering conditions, the purpose of the present study was firstly to evaluate the antinociceptive effects of oral gavage of* T. persicum* both in late phase of formalin test and on sciatic nerve ligated mice and secondly to determine the anti-inflammatory activity of this extract on cotton pellet-induced granuloma formation in mice.

## 2. Materials and Methods

### 2.1. Animals

In this study, all animal manipulations were carried out according to the Helsinki Convention. The subjects used in this study were male albino mice (25–30 g) from the Faculty of Pharmacy, Zabol University of Medical Sciences, Zabol, Iran. All animals were housed in groups of five per stainless-steel cages in a well-ventilated room and allowed to adapt to their environmental controlled conditions (25 ± 2°C and 12 : 12 hours light-dark cycle) before and during the experiments. They were having free access to food and water. All animal experiments were done during the light cycle.

### 2.2. Drugs

Morphine (Daroupakhsh Co.), Imipramine (Sobhan Darou Co.), and Diclofenac sodium (Daroupakhsh Co.) were dissolved in saline and were injected intraperitoneally (i.p.). Ketamine (Alfasan, Holland) and Xylazine (Pantex Holland B.V.) were employed for surgical anesthesia.

### 2.3. Plant Material and Preparation of Aqueous Extract

Twigs and leaf of* Teucrium persicum* Boiss. at the maturing stage were collected from around of Lar, Iran, and chopped. Sample was dried at room temperature in the shade. The taxonomy was confirmed by the Central Herbarium of Medicinal Plants, Iran, and a voucher specimen (number 397) was deposited in the herbarium. Aerial parts of plants (100 gr) were extracted with water, using percolation. The extract (12.5 gr) was concentrated by rotary evaporator. The crude* T. persicum* extract (TPE) was subjected to test.

### 2.4. Dose Determination

Since there was no study on this plant, at first we determined lethal dose. After assays dose of 800 mg/kg was lethal; therefor doses of 100, 200, and 400 mg/kg TPE were selected. After the formalin test for significant differences between the three doses 100, 200, and 400 mg/kg (^*∗∗∗*^ *P* < 0.001), dose of 400 mg/kg as the maximum dose and doses of 100 and 200 mg/kg were selected as lower doses.

### 2.5. Antinociceptive Assays

#### 2.5.1. Formalin Test

A total of 48 male mice in appropriate weight range were selected and randomly were allocated to 6 groups equally. Three groups received the TPE at different doses (100, 200, and 400 mg/kg) orally gavaged for one week. Control group received the carrier of the TPE by oral gavage for one week. Other two groups received single doses of Diclofenac (10 mg/kg) and Morphine (9 mg/kg) intraperitoneal injection 30 minutes before subcutaneous injection of 20 *μ*L of 0.5% formalin into the right dorsal hind paw of the mouse, respectively. The licking time (LT) and the licking number (LN) of the right hind paw by the mouse were recorded for acute phase (0–5 min) and chronic phase (16–60 min) after the formalin injection. In this test, Morphine and Diclofenac were used as positive controls.

#### 2.5.2. Writhing Test

A total of 64 male mice in appropriate weight range were selected and randomly were allocated to 8 groups equally. Three groups received TPE by oral gavage at doses of 100, 200, and 400 mg/kg one hour before the test and also 0.6% acid acetic was injected intraperitonealy a half hour before test. Also a group as control received the carrier of the TPE by oral gavage one hour before the test. Other groups received intraperitoneal injection of a single dose of Morphine (9 mg/kg), Diclofenac (10 mg/kg), Morphine + Naloxone (0.4 mg/kg), and dose of 400 mg/kg TPE + Naloxone (0.4 mg/kg), respectively.

#### 2.5.3. Tail Jump Test

A total of 40 male mice within the appropriate weight were selected and randomly were allocated to 5 groups: there were 8 mice intact male in each group. Three groups received the TPE by oral gavage at doses of 100, 200, and 400 mg/kg one hour before the test. Also a control group received the carrier of the TPE by gavage and other control group received a single dose of Morphine (9 mg/kg) by intraperitoneal injection a half hour before the test. Mice tail from a distance of 20 mm from the junction to the body were placed on the light part in each group. Cut-off time of 20 seconds was considered and waving tail and trawling were scale of animal's response to light beam.

#### 2.5.4. Hot-Plate Test

Pain sensitivity in sciatic nerve ligated mice (a model of neuropathic pain) was evaluated using the hot-plate test as described in previous studies with minor modifications [30]. At first, animals were anesthetized with Ketamine (80 mg/kg) and Xylazine (20 mg/kg) and then the animal's right sciatic nerve was ligated by a copper wire based on the method of Seltzer et al. 1990 [31]. All nerve ligated animals received TPE (100, 200, and 400 mg/kg) for 14 consecutive days via gavage needles once a day. Latency to licking and lifting paws or jumping from the hot-plate surface was determined 14 days after sciatic nerve ligation (cut-off time was restricted on 45 sec). Control animals received saline via gavage needles for the same period of time. Positive control group received Imipramine intraperitoneally at test day.

### 2.6. Anti-Inflammatory Assay

#### 2.6.1. Cotton Pellet-Induced Granuloma Formation in Mice

The details of the cotton pellet-induced granuloma formation were described in previous studies with minor modifications [32, 33]. One sterilized 20 mg adsorbent cotton pellet was implanted subcutaneously in male mice. Animals received* T. persicum* aqueous extract (100, 200, and 400 mg/kg) for 7 days via gavage needles once a day. Control animals received saline via gavage needles for the same period of time. Positive control group received Diclofenac intraperitoneally. On 8th day after cotton pellet implantation, the mouse was sacrificed and the implanted pellet was removed carefully and the wet weight (immediately) and dry weight of the pellet at 60°C (18 h later) were determined. The wet and dry weights of granuloma formulation were calculated.

### 2.7. Statistical Analysis

One-way analysis of variance (ANOVA) followed by Newman-Keuls multiple comparison post hoc test was used for comparison of findings of this study. Unpaired *t*-test was used for comparison between control animals (sham-operated) and the sciatic nerve ligated group. A *P* value of 0.05 or less was considered statistically significant.

## 3. Results

### 3.1. Results of Formalin Test

**Figure 1:**
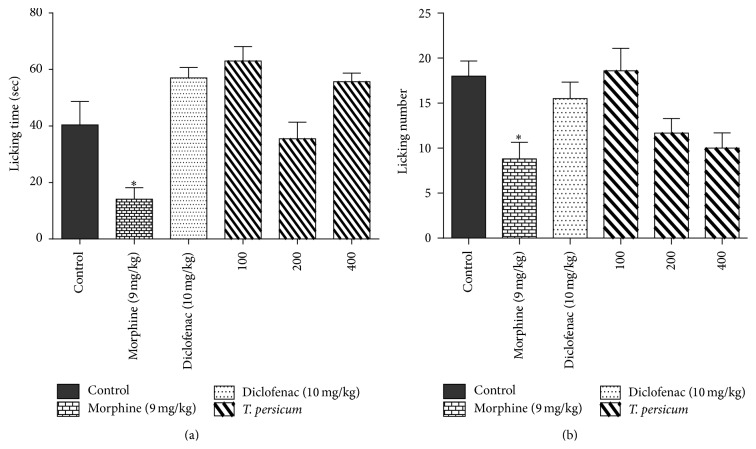
Effects of TPE, Morphine (9 mg/kg), and Diclofenac (10 mg/kg) in the acute phase of the formalin test in mice by assessing the licking time (a) and the number of lickings (b). ^*∗*^ *P* < 0.05, ^*∗∗*^ *P* < 0.01, and ^*∗∗∗*^ *P* < 0.001 significantly different from the control animals. Each value represents the mean ± SEM (*n* = 8).

**Figure 2:**
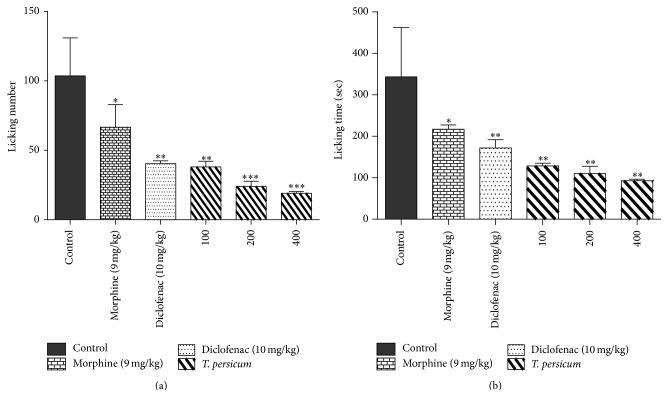
Effects of TPE, Morphine (9 mg/kg), and Diclofenac (10 mg/kg) in the chronic phase of the formalin test in mice by assessing the number of lickings (a) and the licking time (b). ^*∗*^ *P* < 0.05, ^*∗∗*^ *P* < 0.01, and ^*∗∗∗*^ *P* < 0.001 significantly different from the control animals. Each value represents the mean ± SEM (*n* = 8).

As shown in Figure 1, the evaluation of the licking response in the acute phase of the formalin test showed that gavage consecutively of TPE (100, 200, and 400 mg/kg) for 7 days caused a significant decrease on the licking response in comparison with the control groups. There was no significant difference between tested group and control except Morphine (^*∗*^ *P* < 0.05). However in chronic phase, Morphine (^*∗*^ *P* < 0.05) and Diclofenac (^*∗∗*^ *P* < 0.01) exerted significant decrease on the licking response compared to control animals. Also both LT and LN showed a statistically significant difference in the groups receiving the plant extract (100, 200, and 400 mg/kg) compared with control group for LT and LN (^*∗∗*^ *P* < 0.01 for 100 mg/kg for LT and LN; ^*∗∗*^ *P* < 0.01 and ^*∗∗∗*^ *P* < 0.001 for 200 and 400 mg/kg for LT and LN, resp.) (Figure 2).

### 3.2. Results of Writhing Test

**Figure 3:**
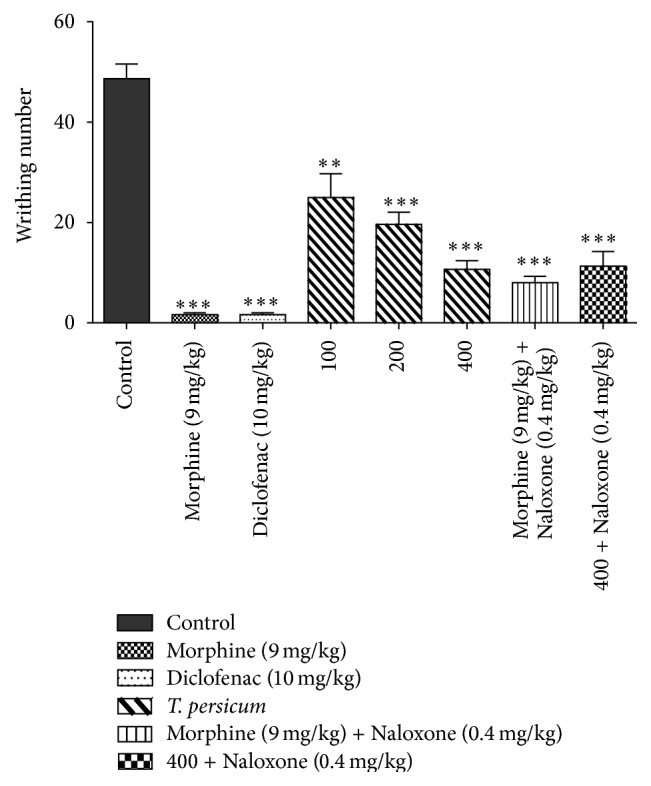
Analgesic effect of TPE in writhing test. ^*∗*^ *P* < 0.05, ^*∗∗*^ *P* < 0.01, and ^*∗∗∗*^ *P* < 0.001 significantly different from the control animals. Each value represents the mean ± SEM (*n* = 8).

The results of writing test showed that all samples except TPE at dose 100 mg/kg (^*∗∗*^ *P* < 0.01) have statistically significant difference (^*∗∗∗*^ *P* < 0.001) gavaged with the control group. Also there is no evidence of statistically significant difference between doses of TPE 400 mg/kg, Morphine + Naloxone, and TPE 400 mg/kg (^*∗∗∗*^ *P* < 0.01). Our study showed that groups receiving Morphine, Diclofenac, dose of 400 mg/kg TPE, dose of 400 mg/kg TPE in combination with Naloxone and Morphine with Naloxone (^*∗∗∗*^ *P* < 0.001), and dose of 200 mg/kg of TPE (^*∗∗∗*^ *P* < 0.01) and dose 100 mg/kg of TPE (^*∗∗*^ *P* < 0.05) showed significant differences compared with the control group (Figure 3).

### 3.3. Results of Tail Jump Test

**Figure 4:**
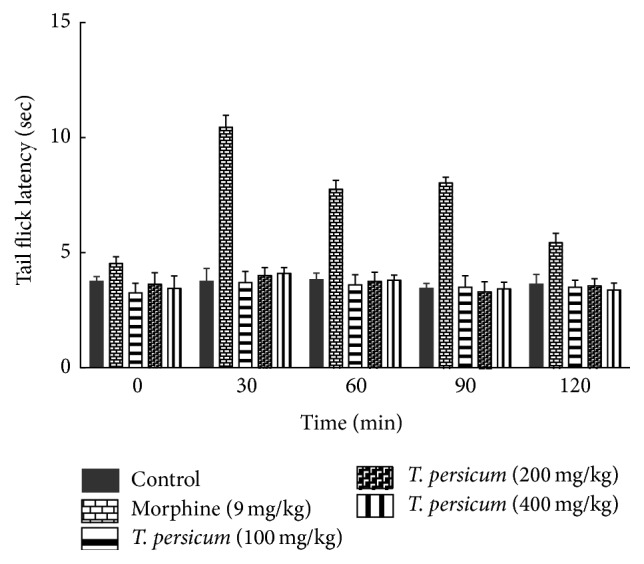
Analgesic effects of plant extract* T. persicum* Boiss. in test tail jump. ^*∗*^ *P* < 0.05, ^*∗∗*^ *P* < 0.01, and ^*∗∗∗*^ *P* < 0.001 significantly different from the control animals (*n* = 8).

The results of our research indicated that there are significant differences between the groups receiving a single dose of Morphine compared to control. Groups receiving plant extract did not show significant difference compared with the control group (Figure 4).

### 3.4. Results of Oral Gavage of TPE in Sciatic Nerve Ligated

**Figure 5:**
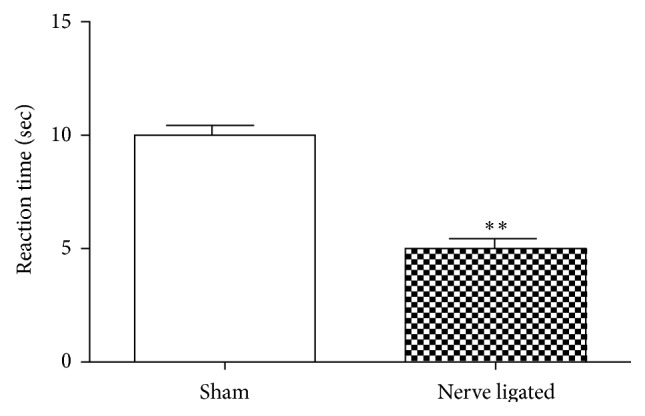
Changes in reaction time in sham-operated and nerve ligated mice, 14 days after the sciatic nerve ligation. ^*∗∗∗*^ *P* < 0.001 significantly different from the control (sham) treated animals. Each value represents the mean ± SEM (*n* = 8).

**Figure 6:**
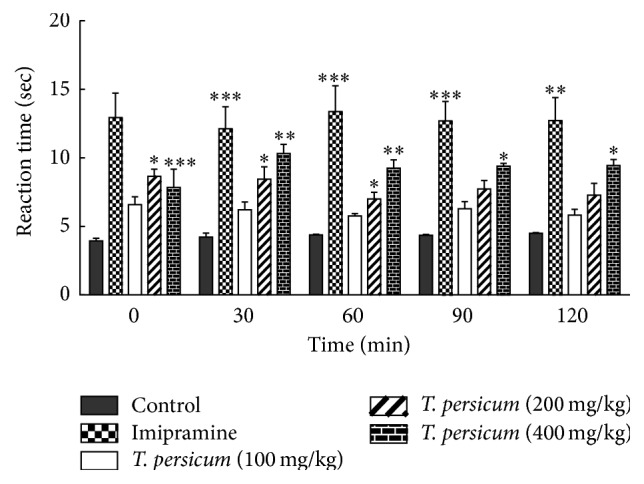
Latency response of the* Teucrium persicum* treated animals in comparison with the control and Imipramine treated animals.. ^*∗*^ *P* < 0.05, ^*∗∗*^ *P* < 0.01, and ^*∗∗∗*^ *P* < 0.001 significantly different from the control animals. Each value represents the mean ± SEM (*n* = 8).

The results showed that there was a significant hyperalgesia between control (sham-operated) and sciatic nerve ligated animals at 14 days after sciatic nerve ligation surgery (Figure 5). As shown in Figure 6, results of 2 week oral gavage of TPE showed that the dose of 100 mg/kg TPE had no significant antinociceptive effect in sciatic nerve ligated animals. Doses of 200 and 400 mg/kg showed significant analgesic effects in sciatic nerve ligated animals. The analgesic effect of TPE (400 mg/kg) remained high until 120 minutes in hot-plate test that was comparable to Imipramine. The analgesic effect of Imipramine as a positive control was started at 30 min and remained high until 120 minutes after the i.p. injection of Imipramine (Figure 6). Findings of our study showed the dose dependent antinociceptive effects of TPE on chronic pain in ligated animals.

### 3.5. Results of TPE in Cotton Pellet-Induced Granuloma Formation in Mice

**Figure 7:**
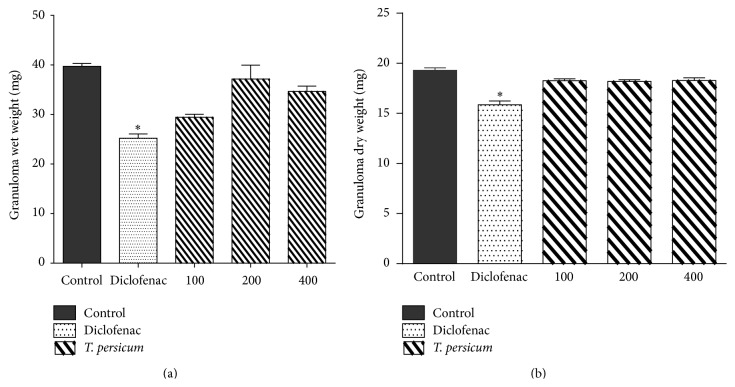
Anti-inflammatory activity of* T. persicum* in cotton pellet-induced granuloma formation. (a) Granuloma wet weight and (b) granuloma dry weight. ^*∗*^ *P* < 0.05, significantly different from the control animals. Each value represents the mean ± SEM (*n* = 8).

All three doses of* T. persicum* extract showed an insignificant anti-inflammatory effect in the cotton pellet-induced granuloma model in mice (Figure 7). However, Diclofenac, at a dose of 10 mg/kg, significantly inhibited the inflammatory responses (^*∗*^ *P* < 0.05).

## 4. Discussion

Results of our study showed that the TPEs have efficient analgesic effects in the chronic phase. Miri et al. [14] reported more than 80 chemical compositions from* T. persicum* that major components were oxygenated monoterpenes and nonoxygenate sesquiterpene (*α*-terpinyl acetate, *α*-cadinene, 1,4-cadinadiene, linalool and cadinol). Also Javidnia et al. [34] identified 81 compounds (93.5% oil) in* T. persicum* such that the main ingredients included caryophyllene oxide,  *α*-pinene, geranyl, linalool, *γ*-cadinene, elemol, and *α*-cadinol. Peana et al. [35] illustrated that linalool has antinociceptive effect. They reported the molecular mechanisms of linalool antinociceptive effect, probably through mechanisms where cholinergic and glutamatergic systems are involved. Ou et al. [36] showed that linalool and 1,8-cineole have analgesic effects through inhibition of prostaglandin and arachidonic acid secretion. Yoon et al. [37] found that *α*-pinene in* Torreya nucifera* has analgesic effects via COX_2_ selective inhibitors which have significant inhibitory effects on PGE_2_. Chavan et al. [38] reported that caryophyllene oxide in* Annona squamosa* L. showed peripheral analgesic effects by inhibition of cyclooxygenase or lipoxygenase. Santos and Rao [39] reported that analgesic effect of 1,8-cineole was related to inhibition of prostaglandins production and cytokine stimulation by monocytes. de Sousa et al. [40] indicated that cadinol monoterpene exerts its analgesic effect by inhibition of prostaglandins synthesis. Miri et al. [13] detected flavonoid compounds in* T. persicum* whose significant antinociceptive activity of flavonoids has been well documented in previous studies [41–44]. Thus, one of the underlying mechanisms of antinociceptive activity of* T. persicum* in chronic pain may be related to its flavonoid source.

The results of our study suggested that TPE has no effect in tail jump test. Therefore, it can be concluded that analgesic effect of the plant is through the central analgesic mechanisms. Finding our study showed that 14 days after sciatic nerve ligation, sensitivity to pain and irritable animals were increased and reaction time to pain and pain tolerance were decreased. So it can be concluded that TPE has proper analgesic effects in model of sciatic nerve ligated in mice (neuropathic pain model).

In our study, Imipramine in mice with sciatic nerve ligated has induced analgesic effects. According to previous studies, tricyclic antidepressants remain one of the first-line therapies for neuropathic pains. Tricyclic antidepressants have specific analgesic actions relating to effects on central nervous system (CNS) monoamines (norepinephrine and serotonin) [45]. Pathophysiological mechanisms of neuropathic pain are not well known. It seems that the spontaneous activity in injured sensory neurons may have a role in this issue.

Still no studies have been done on* Teucrium* that show anti-inflammatory effects of the genus. In our study, TPE did not show a good anti-inflammatory effect in subcutaneous implantation in mice. We proposed that it may be related to dose, method, or period of time used.

## 5. Conclusion

According to TPE analgesic effects that were roughly equivalent with Morphine and with regard to lack of intervention of opioid pathways, it seems that TPE can be a great candidate for drug production with analgesic effects, especially in terms of environmental and chronic nerve pain. We suggested that more studies need to identify mechanisms, analgesic effects of the plant, and pharmacokinetics and pharmacodynamics properties of the extract whereas it is formulated into a form of drug and supplied to the pharmaceutical industries.
